# A Technique for Characterizing the Development of Rhythms in Bird Song

**DOI:** 10.1371/journal.pone.0001461

**Published:** 2008-01-23

**Authors:** Sigal Saar, Partha P. Mitra

**Affiliations:** 1 Department of Biology, The City College of New York, City University of New York, New York, New York, United States of America; 2 Cold Spring Harbor Laboratory, Cold Spring Harbor, New York, United States of America; Columbia University, United States of America

## Abstract

The developmental trajectory of nervous system dynamics shows hierarchical structure on time scales spanning ten orders of magnitude from milliseconds to years. Analyzing and characterizing this structure poses significant signal processing challenges. In the context of birdsong development, we have previously proposed that an effective way to do this is to use the dynamic spectrum or spectrogram, a classical signal processing tool, computed at multiple time scales in a nested fashion. Temporal structure on the millisecond timescale is normally captured using a short time Fourier analysis, and structure on the second timescale using song spectrograms. Here we use the dynamic spectrum on time series of song features to study the development of rhythm in juvenile zebra finch. The method is able to detect rhythmic structure in juvenile song in contrast to previous characterizations of such song as unstructured. We show that the method can be used to examine song development, the accuracy with which rhythm is imitated, and the variability of rhythms across different renditions of a song. We hope that this technique will provide a standard, automated method for measuring and characterizing song rhythm.

## Introduction

Developmental learning (for example, speech acquisition in human infants) takes place early in life but its effects may last the entire lifetime of the individual. Developmental learning is difficult to study because the behavioral changes involved span many time scales: Behavioral changes can occur within hours, across daily cycles of wakefulness and sleep and over developmental stages. The study of developmental song learning in birds provides a unique model system for examining this process in detail.

Previous work has shown that song has structure that spans many time scales [1], [2], [3], [4]. Spectral analysis has proven to be a useful tool in analyzing song temporal structure from milliseconds to several seconds. For example, song spectrograms are the basic tool used to characterize the time-frequency structure of individual songs. Timescales that span several minutes can be analyzed by examining the distribution of syllable features. These distributions reveal stable organized structures (e. g., clusters) even in the early song, where the individual spectrograms appear unstructured. Visual examination of spectrograms and syllable clusters across developmental timescales show the existence of longer time scale structures which have been relatively difficult to quantify.

We find that at these intermediate timescales, it is useful to quantify the rhythmic patterns present in the vocal production, which we call “song rhythm”. There is no accepted method to measure song rhythms in adult song, let alone juvenile song, which appears unstructured and unstable. We show here, how the song rhythm may be extracted by computing spectrograms of time series composed of song features, and that the “rhythm spectrogram” provides a useful tool to characterize and visualize song development over the entire ontogenetic trajectory.

There is a pleasing symmetry between the rhythm spectrogram and the song spectrogram, although the latter exhibits the dynamics of the syringeal apparatus and the song system, while the former exhibits developmental dynamics. In the same way that study of the song spectrograms have led to mechanistic insights into song production at the articulatory and neural system level, we expect that the rhythm spectrogram will provide insight into the developmental dynamics of the nervous system, helping to disentangle genetically driven and environmentally driven effects. For example, do juvenile birds have a steady rhythm prior to song learning? Is the rhythm imitated “as is” or does it evolve from an existing rhythm, etc. More generally, investigating rhythm development can help us understand how birds transform their sensory memory of the song they have heard into a set of complex motor gestures that generate an imitation of that song.

The methods described here are available in the form of MATLAB code distributed as part of the freely available Chronux and Sound Analysis software packages [5], [6].

## Methods

### Glossary of Terms and Units of Analysis

**Figure 1:**
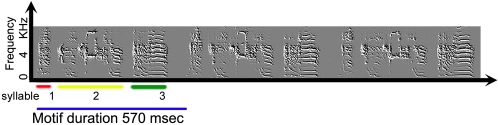
A spectrogram of an adult zebra finch song. This song has three repetitions of the motif. An occurrence of song is called a bout.

The song **bout** is composed of introductory notes followed by a few renditions of a song **motif**. A **syllable** is a continuous sound [7], [8], [9] bracketed by silent intervals. In this paper we define the **motif duration** as the duration of the syllables and silent intervals, including the silent interval after the last syllable as measured in a song with more than one motif. Figure 1 displays an example of a bout with three motifs where each motif has three syllables.

### Multitaper spectral analysis

We make use of the multitaper framework of spectral analysis [10], [11]. In addition to robust estimates of spectra and dynamic spectra for signals with complex structure, the multitaper framework also provides robust estimates of time and frequency derivatives of the spectrogram, which we use as the starting point for the computations of song features other than amplitude [12].

### Recording and Analysis

#### Subjects & training

We used 48 zebra finches (Taeniopygia guttata) from the City College of New York breeding colony. All birds were kept in social isolation from day 30 to day 90 after hatching. Twelve birds were kept in social isolation and were not exposed to conspecific songs. 36 birds were trained starting from day 43 after hatching with one of three different song playbacks (twelve birds per song model) [13], [14]. The number of playbacks was limited to 10 playbacks per session, two sessions per day. The playbacks were initiated by key pecking. Speakers were placed behind a bird model at the far edge of the cage. Birds were raised from hatching under an artificial photoperiod of 12 h : 12 h LD.

#### Data acquisition

To facilitate the acquisition and analysis of the continuous recording of song development of individual birds, we have developed an open source software program that automates much of the data acquisition, feature calculation and database handling-Sound Analysis Pro. Song activity was detected automatically and saved (16 bits, sampling frequency 44.1 kHz) continuously throughout the experiment, except when song playbacks were played. We recorded and analyzed 10 terabytes of song, stored as wave files in Lacie external HDs. Songs were analyzed with the batch module of Sound Analysis Pro, and results (for example, millisecond features) were stored in mySQL 4.0 tables (http://mySQL.com). The batch module did spectral analysis and computation of acoustic features using the first and second taper of multitaper spectral analysis [10], [11] to compute spectral derivatives and acoustic features [5]. Subsequent analysis was based on the six acoustic features computed on each spectral frame: amplitude, pitch, entropy, FM, continuity and goodness of pitch [12]. Those features were computed using a 9.27ms advancing in steps of 1ms, effectively smoothing the data with 89.2% overlap. Final stages of analysis were performed with MATLAB (The Mathworks, Natick, MA).

### Preliminary Analysis

**Figure 2:**
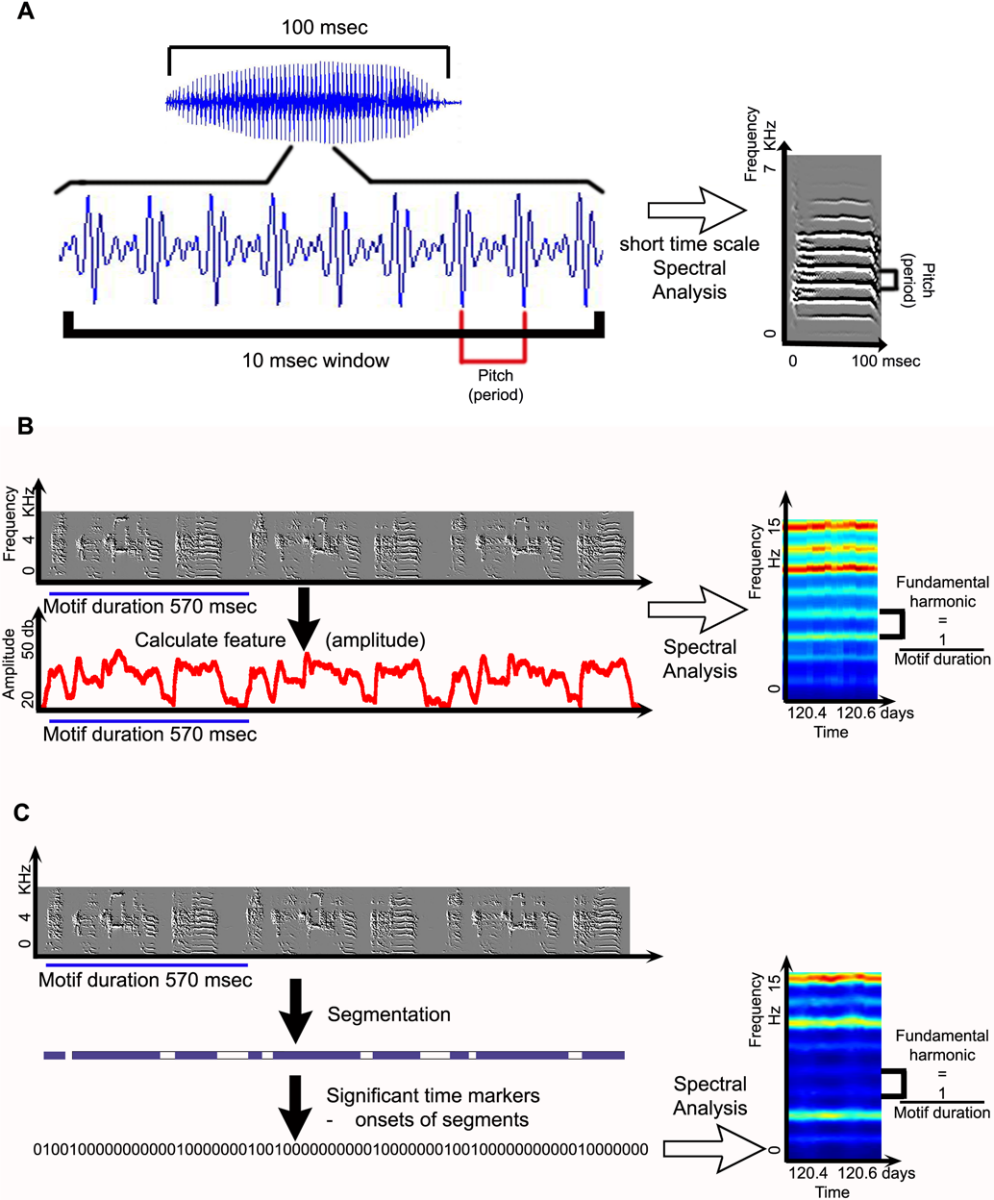
Regular song spectrograms versus Rhythm spectrograms. A. A regular song spectrogram using a 10msec sliding window, showing power up to several kHz. B. Rhythm spectrograms display longer time scales. These are computed by estimating the dynamic spectrum of an appropriate song feature (amplitude in the above example). Each column of the rhythm spectrogram represents the averaged spectrum of song features sung during an hour long interval. C. Rhythm spectrograms that were generated using a point process that marks the onsets of syllables.

The song structure may be summarized using a set of song features such as the amplitude, pitch, mean frequency, amplitude modulation, frequency modulation, continuity in time, continuity in frequency (a definition of these features may be found at [12], [3]). These features summarize the acoustic structure of the song. In addition, a rhythm analysis summarizing the acoustic structure of specific events in the song can be performed. To do this, a “point process” is calculated, a time series in which all values are zero except at the occurrence of an event, where the value is “one”. An event can be notes, syllables or any kind of temporal marker. For example, in figure 2c, “one” marks the onset of a syllable. An amplitude threshold was used to identify the onset of syllables. The threshold was chosen and monitored manually with a graphical user interface.

### Rhythm analysis

The spectrogram, i.e. the short-time spectrum computed with a sliding window, has proven in the past to be a good way of looking at the fine temporal structure of songs [12]. The duration of the sliding window is on the order of 10msec and the spectrum shows power up to several kHz, indicating temporal structure at the millisecond timescale (figure 2A). Analysis of song features has shown temporal structure in the song over longer timescales, including circadian oscillations [2] and developmental song dynamics [13]. One motivation of the current study was to look at these longer timescale dynamics using the same set of tools as was used for the shorter timescale dynamics.

To look at longer time scales, we use a nested spectral analysis method. First, song feature time series are estimated (see 2.2.3 Preliminary Analysis section). The feature values at a given time point depend on the fine temporal structure of the waveform with millisecond resolution, while the features themselves change with a slower timescale of 10–100ms. The continues (not segmented) feature time series are subjected to a second spectral analysis, and the result is a “rhythm” spectrogram, see figure 2B. In the rhythm spectrogram, the fundamental frequency (that was defined as pitch in a normal spectrogram) is in Hz instead of kHz in the regular spectrogram.

Rhythm spectrograms can characterize not only continuous and unsegmented song features, but also point process features where each spike (i.e. a “one”) represents the occurrence of a specific event in the song. We use a point process feature when we want to track how a certain temporal marker develops and how stereotypically it occurs. Those temporal markers could be notes, syllables, or onsets/offsets of syllables. For example, figure 2c shows a feature that marks the onset of syllables.

We were interested in long time scales on the order of an hour, i.e each column in the rhythm spectrogram would correspond to an hour of singing. A time interval of an hour has many bouts of song followed by silent intervals. The analysis is carried out by first segmenting the time period into song bouts and silence. The segmentation to bouts was done using a very low amplitude threshold that was just above noise level. The threshold levels were chosen manually according to the recording quality. We then perform spectral analysis on the feature time series corresponding to each song bout and then average the song bouts that are sung during an hour. By doing so, we are losing the information on temporal structure between bouts, but the spectral structure within a bout remains.

From the rhythm spectrogram, we can derive second order features. In the zebra finch, since the main repeating unit is the motif, the fundamental of the rhythm spectrum may be expected to relate to the motif duration. The degree of periodicity of the rhythm may be assessed in the same way as for the regular song spectrum, using the amplitude and width of the corresponding spectral peaks and the Wiener entropy.

**Figure 3:**
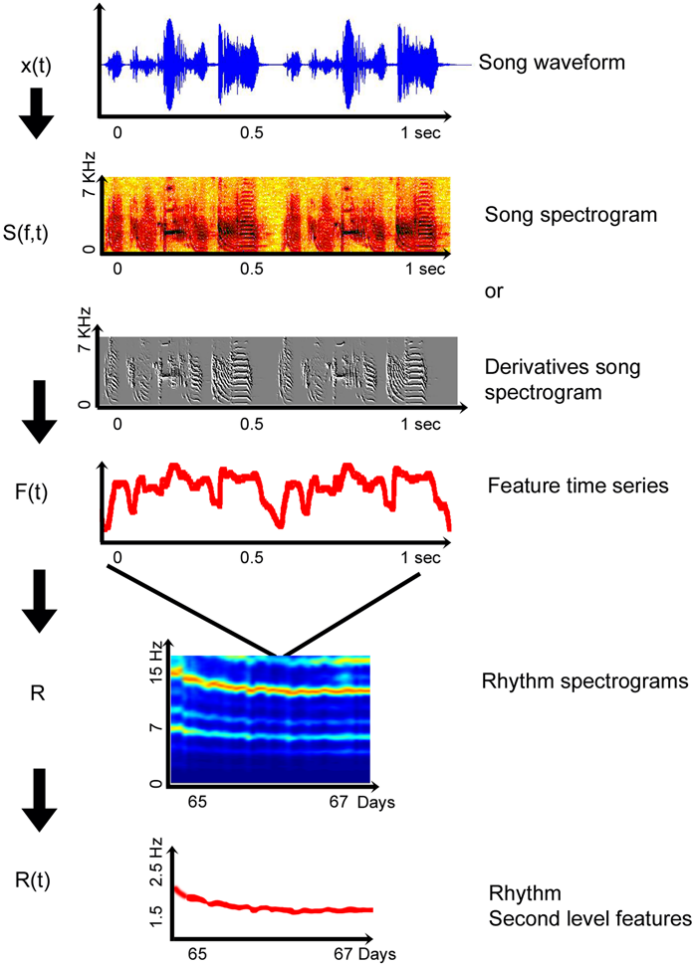
A flowchart of the nested spectral analysis as described in the text.

A flowchart of the procedure is shown in figure 3. The spectrum of the song waveform x(t) is computed to get the song spectrogram S(f,t), or a derivative of that spectrogram [12]. A feature time series F(t) is derived from the spectrogram to get a coarser time scale representation of the song and subjected to a second round of spectral analysis. The result is a “Rhythm” spectrogram S_R_(f,t) which shows the temporal structure on longer (e.g. developmental) time scales. Second level features may be derived from the “Rhythm” spectrograms (e.g. the song rhythm as defined by the fundamental frequency if the spectrogram shows a harmonic structure).

## Results

The adult zebra finch song is composed of a few renditions of the song motif. Each motif has a number of syllables. The rhythm spectrogram shows this repeating structure in the frequency domain, with the fundamental frequency corresponding to the motif duration. In order to verify that this is true, we checked in 20 adult birds, that Indeed, the fundamental of the rhythm spectrograms corresponds to the motif durations. During development there are instances where two types of motifs with two motif durations are sung in one bout, or in different bouts but at the same hour. In those cases, there would be two harmonic trains with different fundamentals. The structure of the harmonics in the rhythm spectrogram, i.e. the energy distribution across the harmonics for one column, is explained by the syllabic structure.

**Figure 4:**
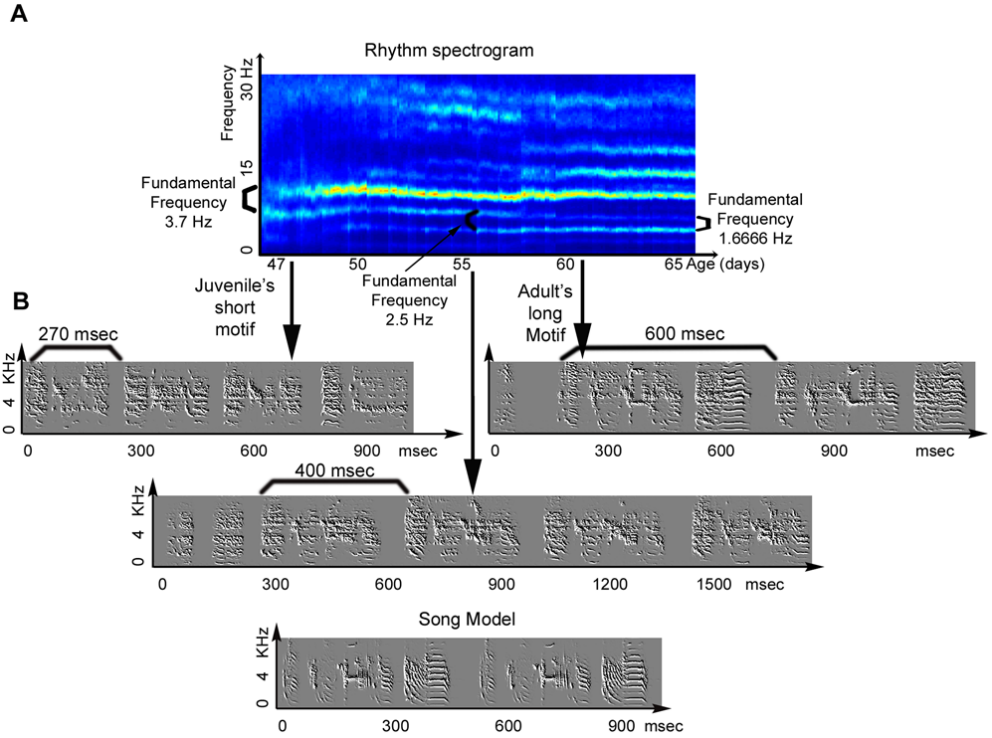
The relations of motif durations and the fundamental frequency of the rhythm spectrogram. Changes in the motif duration show up as changes in the fundamental frequency of the rhythm spectrogram as described in the text.

Figure 4 shows a rhythm spectrum (figure 4a) at a developmental stage where the motif duration changes from 270 ms (3.7 Hz) at the age of 47 days, to 400 ms (2.5 Hz)at the age 55, to 600 ms (1.66 Hz) zHYat age 60 (figure 4b). The transformation from a fundamental of 2.5 Hz to 1.66 Hz, was caused by the incorporation of an additional syllable in the song motif.

Sometimes in low frequencies of the rhythm spectrum it is possible to identify song elements (syllables and notes) that correspond to the rhythm. The energy of the corresponding frequency band increases when either the rhythmic component at that frequency range becomes more periodic or its appearance is more frequent. It is possible to distinguish between these two possible causes by looking at the sharpness of the frequency peak. A signal that is less periodic would appear to be smeared, and a signal that is less abundant will look fainter. For example, the most dominant frequency band (around 11Hz) is caused by a short harmonic stack at the beginning of the motif. At day 47, the energy in that frequency band becomes stronger as the short harmonic stack emerges as a distinct syllable. But, as in sonograms, it is not always straightforward to relate the temporal waveform to the frequency patterns observed in the sonogram. Frequency bands in the rhythm spectrogram might not correspond to syllables and notes in a simple and direct way because rhythm is a global feature of the time varying signal.

**Figure 5:**
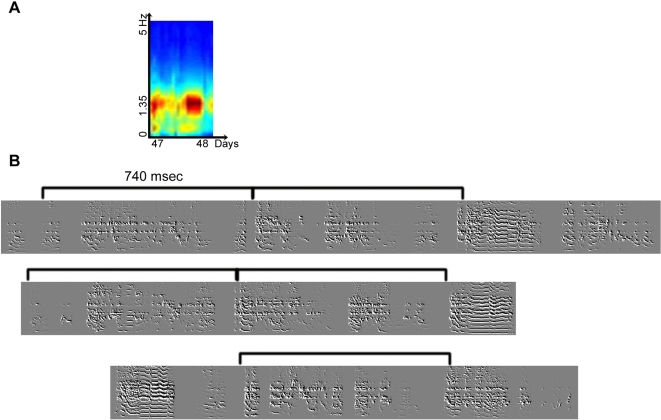
Rhythm of juvenile songs. The rhythm of juvenile song can be identified early during development, as described in the text.

The juvenile's song structure can be highly variable, not only in its notes and syllables, but also in its motif composition (Figure 5B). It is often hard to visually identify a motif, or any repeating unit in the juvenile's song spectrogram. The rhythm spectrogram has proven to be a useful tool in identifying repeated units even in these relatively unstructured songs. Figure 5A shows the rhythm spectrogram for a juvenile bird, age 47–48 days, using the amplitude feature. A strong spectral peak is visible in the rhythm spectrogram at 1.35 Hz. Figure 5B shows a sample of songs from the same days. It is hard to identify by eye any repeating unit in the song spectrogram, but a periodicity of 740msec (corresponding to 1.35Hz) may be found in the onsets of song syllables (highlighted by the black lines- figure 5B).

## Discussion

In this paper we have presented a method that nests spectral analysis across timescales to study longer time scale structure in birdsong development. This technique can detect rhythm early in the zebra finch song development, and can track the transition from the juvenile rhythms to the adult rhythms which correspond to the song motif. The study of rhythm development should provide a different perspective from the one where attention is paid to template matching at the level of the spectral frame [4], [13]. It also promises to provide mechanistic insight into the development of the song circuitry, in the same way that the study of song spectrograms has provided mechanistic insight into the dynamics of the peripheral apparatus that produces song [8], [9].
